# Mary, woman of faith and displaced person: insights for Catholic schools

**DOI:** 10.1007/s40839-021-00156-4

**Published:** 2021-10-15

**Authors:** Stephen J. McKinney

**Affiliations:** School of Education, University of Glasgow, Glasgow, Scotland, UK

**Keywords:** Mary, Catholic schools, Displaced person, Preferential option for the poor

## Abstract

Catholic schools share in the preferential option for the poor that is an essential part of following Jesus and the mission of the Church. Catholic schools in many parts of the world have an historical and contemporary mission for the care and education of the poor. This article uses key passages from the Gospels of Luke and Matthew to illustrate that Mary can be understood as an exemplar of God’s preferential option for the poor. Mary is presented as a young and poor Jewish woman of faith in the Annunciation and the Magnificat in Luke’s Gospel and is presented as an externally displaced person in flight into Egypt in Matthew’s Gospel. The paper also examines the journey to Bethlehem in Luke’s Gospel as interpreted by Pope Francis. He interprets this as Mary being an internally displaced person. Adopting these distinctive modes of interpretation, Mary can be recognised as a model of the preferential option for the poor for Catholic schools.

## Introduction

This article aims to provide some insights for Catholic schools into Mary, the Mother of Jesus, as a woman of faith and as a displaced person. These insights will be derived from some key passages in the Gospels of Luke and Matthew and interpreted within a heuristic framework of the preferential option of the poor. Mary provides an excellent example of God’s preference for the lowly and those who suffer injustice, as expressed, for example, in the Gospel of Luke. De Margerie (1987) explains that this view of Mary is at the centre of the thinking of Victor Codina S J:

Mary personifies the preferential option of God for the Poor, for the weak, and for those who suffer; above all, says Codina, Mary personifies the preference of God toward those who suffer from the injustice of the powerful; she personifies the triumph of God in human weakness and His preference for the humanly insignificant. Let us simply recall here that this is a constant attitude of God in the Biblical History of Salvation. It culminates in God's choice of Mary as Mother of His Son: He chose Mary, a woman of the people, poor and unknown, a virgin (de Margerie, 1987)*.*

Mary is also presented in the Gospel of Matthew as a person who has experienced external forcible displacement—sharing the plight of millions of people in the contemporary world. The article will discuss the interpretation of Pope Francis of the journey to Bethlehem in Luke’s Gospel. He views this as an example of Mary experiencing internal forcible displacement.

The article will begin with a section on the preferential option for the poor and the centrality of this option to Catholic life and practice as espoused within the Church. While acknowledging the origins and roots of the preferential option for the poor, this section will illustrate this with reference to two sources: *A Place at the Table* (2002), a publication of the United States Conference of Catholic Bishops (US Bishops), and some of the writings of Gustavo Gutiérrez. The next section will examine Catholic schools, child poverty and the care for the poor. This will be followed by a lengthy section that will study two aspects of Mary: (1) as woman of faith and (2) as a displaced person. This section will draw on the Annunciation, the Magnificat and the journey to Bethlehem, all from Luke’s Gospel, and the flight into Egypt from Matthew’s Gospel. The article will be completed with some concluding remarks.

## Preferential option for the poor

The phrase ‘preferential option for the poor’, sometimes known as the ‘option for the poor’, that was formulated in Latin America has been used extensively by Liberation Theologians. After a period of serious tension and dispute about some of the theory underpinning Liberation Theology, these two phrases have become mainstream terminology in the Catholic Church and are frequently quoted in publications about Catholic social teaching. They have been used and explored by the last three popes, consolidating the concerns of previous popes for the poor. The preferential option for the poor is a radical option for the poor that is the responsibility of all in the Church (Gutiérrez, 2009a; Sobrino in Powell, 2008). It is rooted in a biblical mandate for care for the poor in both the Old and New Testaments and there is a long history of theological and ecclesial argument for the care of the poor (Groody, 2007). Bartolomé de Las Casas and Gutiérrez, for example, both cite the works of the fourth century theologian St Augustine and his powerful comments on poverty and the neglect of the poor, as an exemplar of the depth of the option in the history of Christian reflection (Dunn, 2013; Grove, 2019; Gutiérrez, 1992).

This preferential option for the poor has been framed as an essential part of Christian discipleship. This emerged in an early form at the second general conference of Latin American Bishops in Medellin in August 1968 and began to be articulated more concretely as the *preferential option for the poor* during the third general conference in Puebla in 1979 and in Liberation Theology. This is emphasised, in a more recent example, by the US Bishops in *A Place at the Table* (2002):

For believers, this mission is not simply a matter of economics or politics but of discipleship. We may sometimes differ about the specifics of how best to serve those in need, overcome poverty, and advance human dignity, but it is impossible for a Christian to say, "This is not my task." This mission is an essential part of what makes us disciples of Christ (US Bishops, 2002, p. VII).

The US Bishops use the analogy of the table: the poor have no place at the table, which is in stark contrast to the practice of Jesus who shared table fellowship with the marginalised and excluded (Lk 15:1–2) (Green, 1995, p. 88). The US Bishops deepen the analogy by referring to the poor being excluded from the table for food *and* the table for decision making. The poor are unable to participate fully in the social, political and economic dimensions of contemporary life. This is unacceptable as the poor are made in the image and likeness of God (as are all people) and as such are ‘endowed with inalienable dignity’ (US Bishops, 2002, p. V). Catholic Christians cannot ignore the face of Christ in the poor:

Although Jesus reminded us that in a world marred by sin, the poor would always be with us (Mtt: 26:11), he also challenged us to see him in those who are hungry or thirsty, in strangers, in the naked and imprisoned. In Christ's description of the Last Judgment, we learn that when we ignore the poor, we ignore Christ himself (Mtt 25:44-45). As John Paul II has declared, "This Gospel text is not a simple invitation to charity. . .. By these words, no less than by the orthodoxy of her doctrine, the Church measures her fidelity."

Gustavo Gutiérrez, one of the leading Liberation Theologians, is equally emphatic that the preferential option for the poor is an essential part of Christian life:

…we see that accompaniment of the poor – so central to the message of the gospel – must always be a reference point for our primary task: following Jesus (Gutiérrez, 2013, p. 32).

This means following the personal example of Jesus who chose a life of poverty, taught about the right use of possessions, and was in solidarity with the poor and the marginalised (Goulder, 1989; Miller, 2014). Gutiérrez argues that the contemporary preferential option for the poor includes a refusal to accept the inevitability of poverty and challenges the causes of poverty (Gutiérrez, 2013, p. 29):

The assumption was that, for some, poverty is the will of God. We cannot accept this. Indeed, to be for the poor is not to accept their poverty. In Liberation theology, we ask how it could be possible to be committed to the poor if we are not against poverty.

Gutiérrez opposes the facile acceptance of a divinely willed poverty which can justify the abrogation of responsibility for the poor. This message of the preferential option for the poor as an essential part of Christian faith and discipleship has been firmly and repeatedly emphasised by Pope Francis and his immediate predecessors (Dorr, 2016; Sedmark, 2016).

## Catholic schools and care for the poor

One of the fundamental principles of a Catholic school, as articulated by some of the key Vatican documents, is that it must have Jesus and the gospel at its heart (Sacred Congregation of Catholic Education 1977; Miller, 2006). There is also an ecclesial dimension to Catholic schools and *The Religious Dimension of the Catholic School* (1988) positions the Catholic school within the Catholic Church (Oldenski, 2011):

The Catholic School finds its true justification in the mission of the Church; it is based on an educational philosophy in which faith, culture and life are brought into harmony (Congregation for Catholic Education, 1988, section 34).

The care of the poor in Catholic schools is stressed in Vatican documents on Catholic education such as *Gravissimum Educationis* (1965), *The Catholic School* (1977) and *Consecrated Persons and their Mission in Schools. Reflections and Guidelines* (2002). These and other Church sources provide many valuable insights into Catholic education and the care for the poor. Nevertheless, it has to be acknowledged that caution must be exercised to avoid the recourse to Catholic ‘edu-babble’ and the eclectic mining for seemingly profound, yet essentially meaningless, phrases such as *Gospel values* (McLaughlin, 1996; Whittle, 2021). Themes need to be explored in depth and layers of meaning and different (legitimate) interpretations need to be uncovered. Catholic schools are called to understand what it means to engage with Jesus, gospel, the mission of the Church, and the contemporary vision and practice of preferential option for the poor in a meaningful way. They can address this by adopting or adapting some of the ideas that have been developed and deepened in research and practice in Catholic schools.

Contemporary research has highlighted the relationship between the preferential option for the poor and Catholic schools in current educational activities and practices in many different parts of the world. This includes Pakistan, India, Africa, United States, South America (Gutiérrez, 2009b; Hrynkow & Creamer, 2015; Kabadi, 2015; McKinney, 2018; Rasiah, 2020, Wodon, 2020; Igbo, 2020). There is body of scholarship that explores the extensive history of Catholic schools being established throughout the world in the 19th to twenty-first centuries with a mission to educate the poor (Marmion, 1984; Grace, 2003; Scanlan, 2008; Barrera, 2019; McKinney & McCluskey, 2019). Further, there is another body of scholarship that discusses the promotion of the preferential option for the poor in the curriculum in Catholic schools, often within teaching on social justice (Grace, 2013; Byron, 2015; Valadez & Mirci, 2015; Admirand, 2018; McKinney, 2019).

### Poverty and child poverty

The discussion on poverty and child poverty in Catholic schools requires a clear understanding of the conception of poverty, the scope of poverty and the impact of poverty on lives and school education. Much of this discussion is, of course, shared with schools that are not Catholic or do not have a mission and rationale rooted within a faith tradition. Contemporary poverty is very complex and child poverty is complicated because children are dependents, and their poverty is intrinsically related to the poverty of the household. Poverty is a multi-faceted societal issue that is, as has been seen, a Christian moral issue, but it is also an issue about moral standards within a society:

The child poverty rate is also a moral standard of what a society is willing to allow children to experience by the accident of their births into particular circumstances, which in many cases, means suffering the deprivation of basic needs by which to grow and come of age, facing diminished opportunities for success, and limited chances for full participation in their society’s growth and development (Chaudry & Wimer, 2016, p. 23).

At this point it would seem appropriate to refer to a series of statistics, preferably official government statistics that use threshold measurements, to reveal the extent of poverty and child poverty in a given context whether it be a city, a local authority/region or a country. These are very useful but blunt measurements that help to map out the scope and the severity of the problem and do provide opportunities to (imprecisely) compare contemporary child poverty to previous eras such as the Victorian period or the harsh economic depression between the two World Wars. These large-scale sets of statistics provide few insights, however, into the reality of poverty and the daily lived experience of deprivation and exclusion (Ridge, 2009). Poverty cannot be simply reduced to numbers, it is about the struggles of individuals to meet their basic human needs with insufficient resource (US Bishops, 2002). There are other complementary ways to measure poverty that are more focussed on the quality of life and the exclusion from the social, cultural and recreational dimensions of life. (Scottish Government, 2021).

The effects of Covid-19 have exacerbated pre-existing effects of poverty and the impact of poverty on school education and have brought some aspects of contemporary child poverty to public attention. These have affected many Catholic schools in the UK, many of which are still located in the more deprived areas, and Catholic schools in many parts of the world. Some notable examples are digital poverty, or digital exclusion, that results in learning loss and food poverty (Wodon, 2021). These aspects of poverty inhibit learning: children are excluded from online learning if they do not have access, or consistent access, to devices and access to the internet, and children are unable to concentrate if they are hungry (McKinney, 2020).

Catholic schools have made strenuous efforts to counter the practical effects of Covid-19 and can also draw on the profound richness of scripture, theology, history of pastoral care, saintly lives and Catholic social teaching that focus on poverty and the Christian response to poverty. As has been mentioned above, there are advocates for promoting Catholic social teaching and the preferential option for the poor in Catholic schools. Gerald Grace (2013), for example, argues for the use of *Caritas in Veritate* (2009) by Pope Benedict XVI as a curricular outline for learning and teaching in Catholic social teaching for Catholic schools. This includes the option for those in poverty, identified as one the seven core principles of Catholic social teaching.

Admirand (2018) proposes that Liberation Theology, and the preferential option for the poor, with the ‘deep moral messages’ and promotion of ‘inclusive, politically engaged and knowledgeable Catholic participation’ should be taught in Religious Education in Catholic schools (pp. 162, 164). He adds that if the teaching is ‘attuned to an interfaith theology’, then, this could be attractive to the children and young people of other denominations and faiths who are enrolled in Catholic schools in many parts of the world (p. 165). Potentially, it would be attractive to those who claim to have no faith or who are disinterested in religion. The teaching of the preferential option for the poor in Religious Education in Catholic schools does raise, however, challenging questions about the configuration of Religious Education, and would not be easily incorporated in a depoliticized and decontextualized understanding and teaching of religion.

There remains scope to enhance and deepen the critical understanding of the preferential option for the poor in Catholic schools and further develop this in the curriculum. One important theme that arguably deserves more attention in the discussion on the preferential option for the poor in Catholic schools is the example of Mary, the Mother of Jesus.

## Mary as woman of faith and displaced person

In the post Vatican II world, Catholic theologians have sought to develop a Mariology that is pertinent for the contemporary church and a return to Mary as represented in scripture and the understanding of Mary in Tradition (Bearsley, 1980; McKenna, 2016, p. 188) highlights three themes for a contemporary ‘synthesis and integration of Mary within Mariology and Theology’. These are:

Mary within Mariology and within theology: Mary, Mother of God, Theotokos, as a Trinitarian reality; Mary, type of the Church, as a Christological reality; Mary in faith and devotion.

McKenna argues that when exploring Mary in faith and devotion we can pose the question: ‘What does Mary’s faith reveal about faith?’ The answer to this question lies in the (limited) information about Mary in the four gospels. She is presented in all of the gospels, though not always necessarily by name (Fitzmyer, 1989, p. 57). John’s Gospel does not name her in the two episodes in his gospel, though these are two crucial passages—the wedding feast of Cana (2:1–12) and her presence at the crucifixion (19:25–27). Mark uses her name only once in the two episodes that feature her in his gospel. Mathew refers to her by name five times in three episodes and refers to her in another episode. The Gospel of Luke furnishes the largest number of references to Mary by name: thirteen times in five episodes and refers to her another three times. Luke also emphasises that Mary was one of the members of the post resurrection community in Acts 1:14 (Bearsley, 1980, p. 473).

This article proposes to examine two aspects of Mary as presented in the gospels: (1) woman of faith and (2) displaced person. This first aspect, woman of faith, will focus on two episodes in Luke’s Infancy Narrative, the rich theological focus on the pre-events and birth of Jesus that inaugurates the ‘renewed relationship between God and humanity’ (Bovon, 2002). These two episodes will be the Annunciation and the Magnificat. In these two episodes, Mary is presented as a young and poor Jewish woman (Krueger, 2019). The second aspect, displaced person, will focus on the journey to Bethlehem for the birth of Jesus in Luke’s Gospel and the flight of the Holy Family into Egypt in Matthew’s Gospel.

### The Annunciation

The angel Gabriel is sent to announce the birth of Jesus to Mary (Lk 1:26–38), having announced the birth of John the Baptist to Zechariah. There are some important contrasts between the announcement to Zechariah and the annunciation to Mary (Krueger, 2019). These help the reader to understand how Mary is represented in Luke’s Gospel. The angel Gabriel, the eschatological messenger, appears to Zechariah in the Temple, the Holy centre of the Jewish world, whereas Gabriel appears to Mary in Nazareth, an un-consecrated space (Green, 1997, p. 84). Mary is not from a privileged background, nor is she as educated as Zechariah, the priest who was allowed to enter the sanctuary of the Temple. The response of Zechariah is presented as ‘hesitant’ and ‘questioning’ (1:18) and ‘rooted in unbelief’ Green (1995, 1997, p. 84). The response from Mary to the announcement by the angel is ‘willing’ and ‘responsive’ (1:29, 38) (Green, 1995). Mary, who has the last word in this episode, as opposed to Zechariah who is silenced in the earlier episode, replies: I am the handmaid of the Lord…Let what you have said be done to me’ (verse 38). The response from the young and poor Jewish woman is exemplary and demonstrates her inner strength and her ‘trusting faith’ (Green, 1997, p. 92; Bovon, 2002, p. 53).

### The encounter with Elizabeth and the Magnificat

Mary hears that her cousin Elizabeth is pregnant and visits her (Lk 1:39–56). In the encounter with Elizabeth, Elizabeth gives a loud cry and says: ‘Of all women you are the most blessed, and blessed is the fruit of your womb’ (1:42) and ‘Yes, blessed is she who believed that the promise made her by the Lord should be fulfilled’ (1:45). Bearlsey (1980) comments that this re-emphasises that Mary has heard the Word of God and has accepted it—the marks of discipleship. She is the first to have faith, to be a believer (Fitzmyer, 1989, p. 69). In response to Elizabeth, Mary proclaims the Magnificat, a canticle. There is some dispute about the origins of this canticle and whether it was borrowed from a Jewish or Jewish Christian group (Balch, 2017). This does not detract from the important function of the canticle:

Canticles add a meta-syntax – ‘they break through the confines of space and time …to declare the future outcome and, indeed, the eschatological significance of the events at hand’. Narrative becomes prophecy (Dillon, 2006, p. 460).

This canticle reflects the themes of Hannah in 1 Samuel 2: 1–10 and Deborah’s song in Judges 5 (Carlson, 2018). The Magnificat draws on ‘prophetic themes of liberation, empowerment of the weak, disempowerment of oppressors, and salvation’ and these are expressed in verses 51–55 (Krueger, 2019, p. 106):

He has shown the power of his arm,

He has routed the proud of heart.

He has pulled down princes from their thrones and exalted the lowly.

The hungry he has filled with good things,

The rich sent away empty.

He has come to the help of Israel his servant,

Mindful of his mercy.

According to the promise he made to our ancestors –

Of his mercy to Abraham and to his descendants forever.

The reversal of the mighty being cast down and the hungry filled with good things anticipates the passage read by Jesus in the synagogue (Luke 4:18–19) and the Beatitudes (6:20–26) (Latin American Episcopate, 1979; Knight, 1998, p. 74). It is unsurprising that Latin American Liberation Theology views the Magnificat as a striking example of the preferential option for the poor. The Latin American Episcopate in the *Puebla Final Document* (1979) highlight the links between the Old and New Testaments in the Magnificat:

The Magnificat mirrors the soul of Mary. In that canticle we find the culmination of the spirituality of Yawheh’s poor and lowly, and of the prophetic strain in the Old Testament. It is the opening proclamation of Christ’s new Gospel.

The verses of the Magnificat have been interpreted in different ways. Some scholars have interpreted them metaphorically as referring to spiritual hunger and spiritual poverty and this is critiqued as a ‘depoliticizing spiritualization of the Magnificat and Mary’ (Balch, 2017, p. 655). Other scholars have interpreted the verses as referring to the concrete hunger and poverty that Mary would have fully understood in her position in the lower end of the social strata (Gutiérrez, 1971). The verses of joy and thanksgiving at the beginning of the Magnificat are closely connected to the later verses and the ‘action of God who liberates the oppressed and humbles the powerful’ (Gutiérrez, 1971, pp. 207–208). In a subsequent work, Gutiérrez provides an insight into the integral connection between the faith of Mary, as expressed in the Magnificat, and the practical consequences of that faith in the option for the poor:

The canticle of Mary combines a trusting self-surrender to God with a will to commitment and close association with God’s favourites: the lowly, the hungry (Gutiérrez, 1983, p.127).

The preferential option for the poor in the canticle is affirmed as an essential component of the relationship with God.

### Mary as displaced person in the Gospel of Luke

There are around 82.4 million people forcibly displaced in the contemporary world (UNHCR, 2021). People are displaced because of persecution, conflict and war, violence and violations of human rights. The forcibly displaced includes refugees and asylum seekers who flee to external sites, to other countries, and also includes internally displaced persons. People who are internally displaced are displaced within their own country and constitute over half of those who experience forcible displacement. According to the Gospels of Luke and Matthew, Mary experienced both internal and external forcible displacement. She experienced internal displacement before the birth of Jesus (Lk: 2:1–5) and external displacement after his birth (Matt: 2:13–18).

Pope Francis focused on the journey of Mary and Joseph from Nazareth to Bethlehem, in his sermon on the Nativity of the Lord on the 24th December 2017. In his sermon, he framed this journey as an example of the bewildering and frightening internal displacement that is experienced by many families in the contemporary world.

They had to leave their people, their home and their land, and to undertake a journey in order to be registered for the census. This was no comfortable or easy journey for a young couple about to have a child: they had to leave their land. At heart, they were full of hope and expectation because of the child about to be born; yet their steps were weighed down by the uncertainties and dangers that attend those who have to leave their home behind. (Pope Francis, 2017).

There was movement within Palestine at this period of time for religious festivals but that was movement that was willingly undertaken. In this instance of movement, Joseph and the heavily pregnant Mary (the birth of Jesus is imminent) have been forced to make a journey through the pressures of a tyrannical external party and have left the safe space of their home and the support of family and friends to enter a new unknown space. This is what Codina means when he states that ‘Mary personifies the preference of God toward those who suffer from the injustice of the powerful’ (de Margerie, 1987)*.* When they arrived in Bethlehem they were confronted with further challenges:

Then they found themselves having to face perhaps the most difficult thing of all. They arrived in Bethlehem and experienced that it was a land that was not expecting them. A land where there was no place for them. And there, where everything was a challenge, Mary gave us Emmanuel (Pope Francis, 2017).

This appealing and intriguing framing by Pope Francis is highly consistent with the strong emphasis that he places on the responsibility of the Church towards refugees and migrants (Guzik, 2018).

### Mary as displaced person in the Gospel of Matthew

The external displacement occurred after Jesus was born. In the narrative in Matthew that recounts the flight into Egypt, the Angel of the Lord appears to Joseph (coherent with his more prominent role in the Matthew infancy narrative) and warns him that he must flee the threat of Herod and take the family to the safer destination of Egypt (2:13) (Dube, 2019; Eurell, 2020). There are, of course close parallels with the Joseph and Moses stories and the Exodus narrative. The flight into Egypt is often interpreted as Jesus being a refugee at an early age, as he flees with his family. This early experience is compared with the experience of externally forcibly displaced people throughout the world (Houston, 2015; Korgen & Gallagher, 2013). Joseph and Mary, as new parents, also experience this forcible displacement and flee in haste to Egypt as refugees, Mary nursing a newly born baby. Joseph and Mary, like many forcibly displaced persons try to protect their family and, in particular, the youngest member of the family and lead them to safety.

## Concluding remarks

The article has stressed that Catholic schools share the biblical, theological and ecclesial mandate of the preferential option for the poor. As Catholic schools understand and enact this preferential option, the paper has offered the example of Mary and has interpreted some gospel passages that feature her by using the preferential option for the poor. This is a distinctive approach that is discordant with a depoliticized and decontextualized understanding and teaching of the highlighted passages in the article. This approach is not the only way to interpret these passages, nor is the study of Mary in the gospels the only way to understand the position of Mary in the Catholic Church. This approach to the experiences of Mary does enable an understanding of the faith of this young and poor Jewish girl, and the experience of Mary as displaced person that resonates with the experiences of many people throughout the world who suffer the indignity of poverty and the anguish and pain of internal and external forcible displacement.

## References

[CR1] Admirand P (2018). Why liberation theology should be taught in Catholic secondary schools. International Studies in Catholic Education.

[CR2] Balch DL (2017). Mary’s Magnificat (Luke 1:46b–55) and the Price of Corn in Mexico. Journal of Biblical Literature.

[CR3] Barrera A (2019). Catholic missionaries and their work with the poor. Mitigating market-government failure in emerging nations.

[CR4] Bearsley PJ (1980). Mary the perfect disciple: A paradigm for mariology. Theological Studies.

[CR5] Bovon, F. (2002). *Luke 1. A Commentary of the Gospel of Luke 1:1–9:50*. (Christine M. Thomas, Trans.). Fortress Press.

[CR6] Byron WJ (2015). What Catholic schools can do about world hunger. International Studies in Catholic Education.

[CR7] Carlson R (2018). Hannah at Pentecost. On Recognizing Spirit Phenomena in Early Jewish Literature. Journal of Pentecostal Theology.

[CR8] Chaudry A, Wimer C (2016). Poverty is not just an indicator: The relationship between income, poverty, and child ewell-being. Academic Pediatrics.

[CR9] Congregation for Catholic Education (1988). The religious dimension of education in a Catholic school.

[CR10] Congregation for Catholic Education (2002). Consecrated Persons and their mission in schools. Reflections and guidelines.

[CR11] de Margerie B (1987). Mary in Latin American liberation theologies. Marian Studies.

[CR12] Dillon RJ (2006). The benedictus in micro- and macrocontext. The Catholic Biblical Quarterly.

[CR13] Dorr D (2016). Option for the Poor and for the Earth. From Leo XIII to Pope Francis.

[CR14] Dube Z (2019). Jesus—The Immigrant Egyptian Jews in Matthew’s Sonergut: A Migration perspective. HTS Theological Studies.

[CR15] Dunn GD (2013). Augustine’s homily on almsgiving. Journal of Early Christian History.

[CR16] Eurell J-C (2020). Mariological developments in the new testament. Horizons in Biblical Theology.

[CR17] Fitzmyer JA (1989). Luke the theologian. Aspects of his teaching.

[CR18] Goulder MD (1989). Luke a new paradigm 1.

[CR19] Grace G (2003). ‘First and foremost, the Church offers its educational service to the poor’: Class, inequality and Catholic schooling in contemporary contexts. International Studies in Sociology of Education.

[CR20] Grace G (2013). Catholic social teaching should permeate the Catholic secondary school curriculum: An agenda for reform. International Studies in Catholic Education.

[CR21] Green JB (1995). The theology of the Gospel of Luke.

[CR22] Green JB (1997). The Gospel of Luke.

[CR23] Groody DG, Groody DG (2007). Introduction. The option for the poor in Christian theology.

[CR24] Grove K (2019). Rhetoric and reality: Augustine and Pope Francis on preaching Christ and the poor. Theological Studies.

[CR26] Gutiérrez G (1971). A theology of liberation.

[CR27] Gutiérrez G (1983). We drink from our own wells.

[CR28] Gutiérrez, G. (1992). *La Casas In search of the poor of Jesus Christ.* (Robert Barr, Trans.). Orbis Books.

[CR29] Gutiérrez G (2009). The option for the poor arises from faith in Christ. Theological Studies.

[CR30] Gutiérrez G, Griffin M, Block JW (2013). Saying and showing to the poor “God Loves you”. In the Company of the Poor. Conversations with Dr. Paul Framer and Fr. Gustavo Gutiérrez.

[CR25] Gutiérrez AL (2009). The preferential option for the poor in Catholic education in the Philippines: A report on progress and problems. International Studies in Catholic Education.

[CR31] Guzik P (2018). Communicating migration—Pope Francis’ strategy of reframing refugee issues. Church, Communication and Culture.

[CR32] Houston FS (2015). You shall love the stranger as yourself.

[CR33] Hrynkow C, Creamer D (2015). Catholic social thought bearing fruit: The case of Sister Cyril Mooney and Loreto Day School Sealdah, Kolkata, India. International Studies in Catholic Education.

[CR34] Igbo, M. U. R. (2020). *Catholic schools and the interests of the poor in Nigeria*. Doctoral Thesis. University College, London.

[CR35] Kabadi SU (2015). The Jesuit social justice dialectic within the Cristo Rey School model. Journal of Catholic Education.

[CR36] Knight J (1998). Luke’s Gospel.

[CR37] Korgen JO, Gallagher VA (2013). The true cost of low prices. The violence of globalization.

[CR38] Krueger L (2019). Reinterpreting Eve and Mary. Pneuma.

[CR39] Eagleson J, Scharper P, Latin American Episcopate (1979). Evangelization in latin america’s present and future. Final Document of the third general Conference of the Latin American Episcopate, Puebla de Los Angeles, Mexico. Puebla *and Beyond*.

[CR40] Marmion J (1984). The beginning of the Catholic poor schools in England. Recusant History.

[CR41] Miller MJ (2006). The Holy See’s teaching on Catholic schools.

[CR42] McKenna M (2016). New directions in mariology within theology: Mary, Mother of God, theotokos, type of the Church, illuminator of the fullness of Christian faith and theology. Theology.

[CR43] McKinney SJ (2018). The roots of the preferential option for the poor in Catholic schools in Luke’s Gospel. International Studies in Catholic Education.

[CR45] McKinney SJ, Buchanan MT, Gellel A-M (2019). Catholic social teaching, Catholic education and religious education. Global perspectives on Catholic religious education in schools. Volume II: Learning and leading in a pluralist world.

[CR46] McKinney SJ (2020). Covid-19: Food insecurity, digital exclusion and Catholic Schools. Journal of Religious Education.

[CR44] McKinney SJ, McCluskey R (2019). A history of Catholic education and schooling in Scotland: New perspectives.

[CR47] McLaughlin TH, Mclaughlin TH, O’Keefe J, O’Keeffe B (1996). The distinctiveness of Catholic education. The contemporary Catholic School: Context, identity and diversity.

[CR48] Miller AC (2014). Bridge work and seating charts: A study of Luke’s ethics of wealth, poverty and reversal. Interpretation: A Journal of Bible and Theology.

[CR49] Oldenski T (2011). Liberation theology and critical pedagogy in today’s Catholic schools. Social justice in action.

[CR65] Pope Benedict XVI (2009).* Caritas in Veritate*. Retrieved September 8, 2021, from https://www.vatican.va/content/benedict-xvi/en/encyclicals/documents/hf_ben-xvi_enc_20090629_caritas-inveritate.html

[CR50] Pope Francis (2017). *Homily of His Holiness Pope Francis. Solemnity of the Nativity of the Lord.*

[CR51] Pope Paul VI (1965). *Gravissimum Educationis (Declaration on Christian Education).* Retrieved September 8, 2021, from http://www.vatican.va/archive/hist_councils/ii_vatican_council/documents/vat-ii_decl_19651028_gravissimum-educationis_en.html

[CR52] Powell R (2008). Theology in public reason and legal discourse: A case for the preferential option for the poor. 15 Wash and Lee. Journal of Civil Rights and Social Justice.

[CR53] Rasiah SJ (2020). Jesuit schools in the archdiocese of Lahore, Pakistan: A service for the poor. International Studies in Catholic Education.

[CR54] Ridge, T. (2009). *Living with poverty. A review of the literature on children’s and families’ experience of poverty*. Department of Work and Pensions. Research Report No 594.

[CR55] Sacred Congregation for Catholic Education (1977). The Catholic school.

[CR56] Scanlan M (2008). The grammar of Catholic schooling and radically “Catholic schools”. Catholic Education: A Journal of Inquiry and Practice.

[CR57] Scottish Government. (2021). *Child material deprivation.* Retrieved September 8, 2021, from https://www.nationalperformance.gov.scot/child-material-deprivation

[CR58] Sedmark C (2016). A church of the poor. Pope Francis and the transformation of orthodoxy.

[CR59] UNHCR (2021). *Figures at a Glance.* Retrieved September 8, 2021, from https://www.unhcr.org/uk/figures-at-a-glance.html

[CR60] United States Conference of Catholic Bishops. (2002). *A place at the table.* Retrieved September 8, 2021, from https://www.usccb.org/resources/place-table

[CR61] Valadez JR, Mirci PS (2015). Educating for social justice: Drawing from Catholic social teaching. Journal of Catholic Education.

[CR62] Whittle S, Whittle S (2021). Moving beyond slogan and piety: The real challenge facing researchers developing the philosophy and theology of Catholic education. Irish and British reflections on Catholic education.

[CR63] Wodon Q (2020). How well do Catholic and other faith-based schools serve the poor? A study with special reference to Africa: Part II: Learning. International Studies in Catholic Education.

[CR64] Wodon Q (2021). Global Catholic Education Report 2021. Education, pluralism, learning poverty and the right to education.

